# Exploring the mechanism underlying hyperuricemia using comprehensive research on multi-omics

**DOI:** 10.1038/s41598-023-34426-y

**Published:** 2023-05-03

**Authors:** Hengrui Liu, Ruolin Xie, Qiongqiong Dai, Ji Fang, Yunbo Xu, Bo Li

**Affiliations:** 1 Shenzhen Baoan Authentic TCM Therapy Hospital, Shenzhen, China; 2 Xinkaiyuan Pharmaceuticals, Beijing, China; 3 Guangzhou Institutes of Biomedicine and Health, Chinese Academy of Sciences, Guangzhou, China; 4 Tianjin Yinuo Biomedical Co., Ltd, Tianjin, China

**Keywords:** Genetics, Biomarkers, Diseases, Pathogenesis, Risk factors, Urology

## Abstract

Hyperuricemia involves multiple complex metabolisms, but no study has conducted a comprehensive analysis using human blood and urine metabolomics for hyperuricemia. Serum and urine samples from 10 patients with hyperuricemia and 5 controls were collected and analyzed by the UHPLC-MS/MS. Differential metabolites were identified and used in the enrichment analysis where we collected hyperuricemia target genes. Hyperuricemia kidney differential expressed genes (DEGs) were identified using RNA-sequencing data from the hyperuricemia mouse model induced by the potassium oxonate. A Mendelian randomization analysis of the association between caffeine-containing drinks and gout risk was conducted. An intersection analysis between hyperuricemia target genes and hyperuricemia kidney DEGs was conducted and the resulting genes were used for network analysis using the STRING. 227 differential metabolites were identified as differential metabolites and were enriched in 7 KEGG pathways, among which “Caffeine metabolism” was the top. The Mendelian randomization analysis revealed a significant association between tea or coffee intake and gout risk. There were 2173 genes that were identified as hyperuricemia kidney DEGs from mouse data. The intersection analysis identified 51 genes for the hyperuricemia regulation network. A hyperuricemia regulation protein network in the kidney was constructed. This study suggested a potential association between caffeine and hyperuricemia and constructed a hyperuricemia regulation network for future reference.

## Introduction

Hyperuricemia is a disease manifested by a prolonged pathophysiological high level of uric acid in the blood. Patients are diagnosed to have hyperuricemia when their serum uric acid is over 420 µmol/L (7.0 mg/dL)^1,2^. This disease results from a pathophysiological drop in intestinal or renal excretion of urate or a pathophysiological raise of hepatic biosynthesis^3^. Normally, the source of urate in the human body is the enzymatic degradation of nucleotides or purine nucleobases. This biochemical process involves the formation of RNA and DNA and energy metabolism^4^. As the terminal metabolite of purine catabolism, urate is not used for the synthesis of GTP or ATP^5^. For this reason, an excessive intake of food with rich purine, such as seafood and red meat, can induce an aberrantly high level of blood urate^6^. Other potential factors for hyperuricemia include alcohol and fructose intake^7,8^. Therefore, the incidence of this disease is closely related to the local economic conditions, which determine the type of food that people intake. This might explain why hyperuricemia has a higher prevalence in developed countries than in underdeveloped countries as well as the increased rate of hyperuricemia in developing countries over time^9^. Worse, hyperuricemia often comes with several comorbidities, such as diabetes mellitus, cardiovascular diseases, hypertension, and hyperlipidemia. Therefore, hyperuricemia is one of the largest public health issues and will become more widespread in the population as the global economy continues to develop.

Hyperuricemia is often heritable, which suggests the importance of genetic contributions to this disease^10^. This indicates that, besides the aforementioned environmental factors, genetic factors might contribute to the regulation of urate homeostasis. An example is the Lesch–Nyhan syndrome where genes that cause the dysfunction of critical enzymes responsible for purine biotransformation can lead to hyperuricemia^11^. Medical intervention can regulate urate homeostasis. For instance, allopurinol, a xanthinoxidase inhibitor, is used for hyperuricemia patients which can inhibit urate biosynthesis^12^. In the clinic, patients with hyperuricemias caused by over-biosynthesis of urate are less than 10% of the total hyperuricemia patients, whereas more than 90% of hyperuricemias result from a decreased ability of the kidney or intestine in the urate excretion^13,14^. Data suggested that approximately 70% of urate is eliminated by the kidney glomerulus^15^, hence, most of the studies believed that the renal proximal tubule cell is the primary impact factor and a potential target cell for hyperuricemia. In the kidney, the renal proximal tubule cells express several transporters that can excrete urate or reabsorb urates, such as URAT1, GLUT9, NPT1, NPT4, OAT1, and OAT3^4,16–21^. Therefore, target therapies derived from this knowledge have been used for clinical hyperuricemia, such as benzbromarone, probenecid, and lesinurad^22^. However, modern treatments are unable to guarantee desirable outcomes in clinical hyperuricemia patients. This might be due to the complexity of this disease and our lack of understanding of the genetic regulation of hyperuricemia.

As hyperuricemia involves multiple complex metabolisms, recent studies have applied high-throughput untargeted metabolomics analysis to help understand the mechanism of the disease. Serum from hyperuricemia patients has previously been analyzed with this method^23–25^, while urinary metabolomics has been used to evaluate the effect of hyperuricemia on the kidney in a mouse model^26^. To date, no study has jointly analyzed human blood and urine in metabolomics for hyperuricemia. In this study, we solved this puzzle by using metabolomics analysis in human serum and urine, to identify potential gene targets of hyperuricemia in the kidney. The combined analysis of serum and urine enables a comprehensive observation of the difference in metabolisms. We also jointly analyzed gene expression data of the hyperuricemia mouse model. Eventually, we constructed a hyperuricemia regulation network using human serum, urinary metabolomics, and a mouse model. We believe that this study contributes to the understanding of hyperuricemia regulation and identifies potential therapeutic targets for future studies. A graphical abstract of this study is provided in the supplementary materials (S-Figure. graphical abstract).

## Methods, subjects, and materials

### Clinical subjects and sample collection

The enrolled participants were male outpatients at the Shenzhen Baoan Authentic TCM Therapy Hospital. Hyperuricemia patients were diagnosed based on the Guideline for the Diagnosis and Management of Hyperuricemia and Gout in China 2019 from the Chinese Society of Endocrinology, Chinese Medical Association^27^. All subjects in the hyperuricemia group had a serum urate level over 420 μmol/l. Participants were not taking any medication that could affect their serum urate level. Participants were excluded if they were diagnosed to have other metabolic diseases. Samples from 15 participants (10 patients with hyperuricemia and 5 controls) were collected. Basic parameters of subjects such as age, creatinine levels, blood urea nitrogen levels, serum uric acid, urine pH, and urine specific gravity were recorded. Venous blood samples and urine samples were collected from the participants at the same time (within 5 min). The blood samples were stabilized for 30 min at 25 °C followed by centrifugation at 3,000 revolutions per minute for 10 min before the supernatants were removed. The serum samples and urine samples were stored at − 80 °C before analysis. The study was approved by the Ethics Committee of the Shenzhen Baoan Authentic TCM Therapy Hospital (ethics reference number: S22C-722112511400), and written informed consent was obtained from all participants. All methods were performed in accordance with the relevant guidelines and regulations.

### Metabolites extraction

The serum or urinary samples (100 μL) were resuspended in 400 μL prechilled 80% methanol, then incubated on ice for 5 min followed by a 20 min centrifugation at 15,000 g at 4 °C. The supernatants were diluted by LC–MS grade water to a final concentration containing 53% methanol. The samples were transferred to another tube followed by another 20 min centrifugation at 15,000 g at 4 °C. The supernatant was analyzed using the LC–MS/MS^28,29^.

### UHPLC-MS/MS analyses

UHPLC-MS/MS analyses were conducted in IGE Biotechnology Co., Ltd. Guangzhou, China with a Vanquish UHPLC system (ThermoFisher, Germany) coupled to an Orbitrap Q Exactive TMHF-Xmass spectrometer (Thermo Fisher, Germany). A Hypesil Gold column (100 × 2.1 mm, 1.9 μm) was used to separate the metabolites. Samples were injected at a flow rate of 0.2 mL/min using a 17-min linear gradient. Eluent A (0.1% formic acid in Water) and eluent B (Methanol) were used as the eluents for the positive polarity mode. Eluent A (5 mM ammonium acetate, pH 9.0) and eluent B (Methanol) were used as the eluents for the negative polarity mode. The solvent gradient was set as follows: 1.5 min 2% B; 3 min 2–85% B; 10 min 85–100% B; 10.1 min 100–2% B; 12 min 2% B. Q Exactive TM HF-X mass spectrometer was operated with a spray voltage of 3.5 kV in positive polarity mode or negative polarity mode. The capillary temperature was set at 320 °C. The sheath gas flow rate was set at 35 psi. The aux gas flow rate was set at 10 L/min. The S-lens RF level was 60. The Aux gas heater temperature was 350 °C.

### UHPLC-MS/MS data processing and metabolite identification

Compound Discoverer 3.1 (CD3.1, ThermoFisher) was used to process the UHPLC-MS/MS raw data. Data were acquired in the untargeted mode. Peak alignment, peak picking, and quantitation were conducted. The retention time tolerance was set at 0.2 min. The actual mass tolerance was set at 5 ppm. The signal intensity tolerance was set at 30%. The signal/noise ratio was set at 3. The intensity was set at a minimum. The total spectral intensity was used to normalize the peak intensities. The molecular formula was predicted using normalized data according to molecular ion peaks, additive ions, and fragment ions. The mzVault, mzCloud, and MassList databases were used to match these peaks. Statistical analyses were conducted using the Python 2.7.6 version, R version R-3.4.3, and CentOS release 6.6 to obtain the metabolites' identification and relative quantification results.

## Metabolite data analysis

These metabolites were annotated using the MetaboAnalyst tool with the KEGG database, HMDB database, and LIPIDMaps database. Principal components analysis (PCA) and plotting of volcano plot and heatmap were performed with R. A t-test was used to analyze the significance. The metabolites with a variable importance in the projection (VIP) score of ≥ 1 and a *P* value < 0.1 were considered to be differential metabolites. These metabolites were used for the enrichment analysis using the KEGG as well as the HMDB database. The metabolic pathways with a *p* < 0.05 were considered significantly different. The genes in pathways where the hit metabolites are significantly enriched (defined as hyperuricemia KEGG targets) were identified.

## Mouse model and kidney sample RNA sequencing

**Figure 1:**
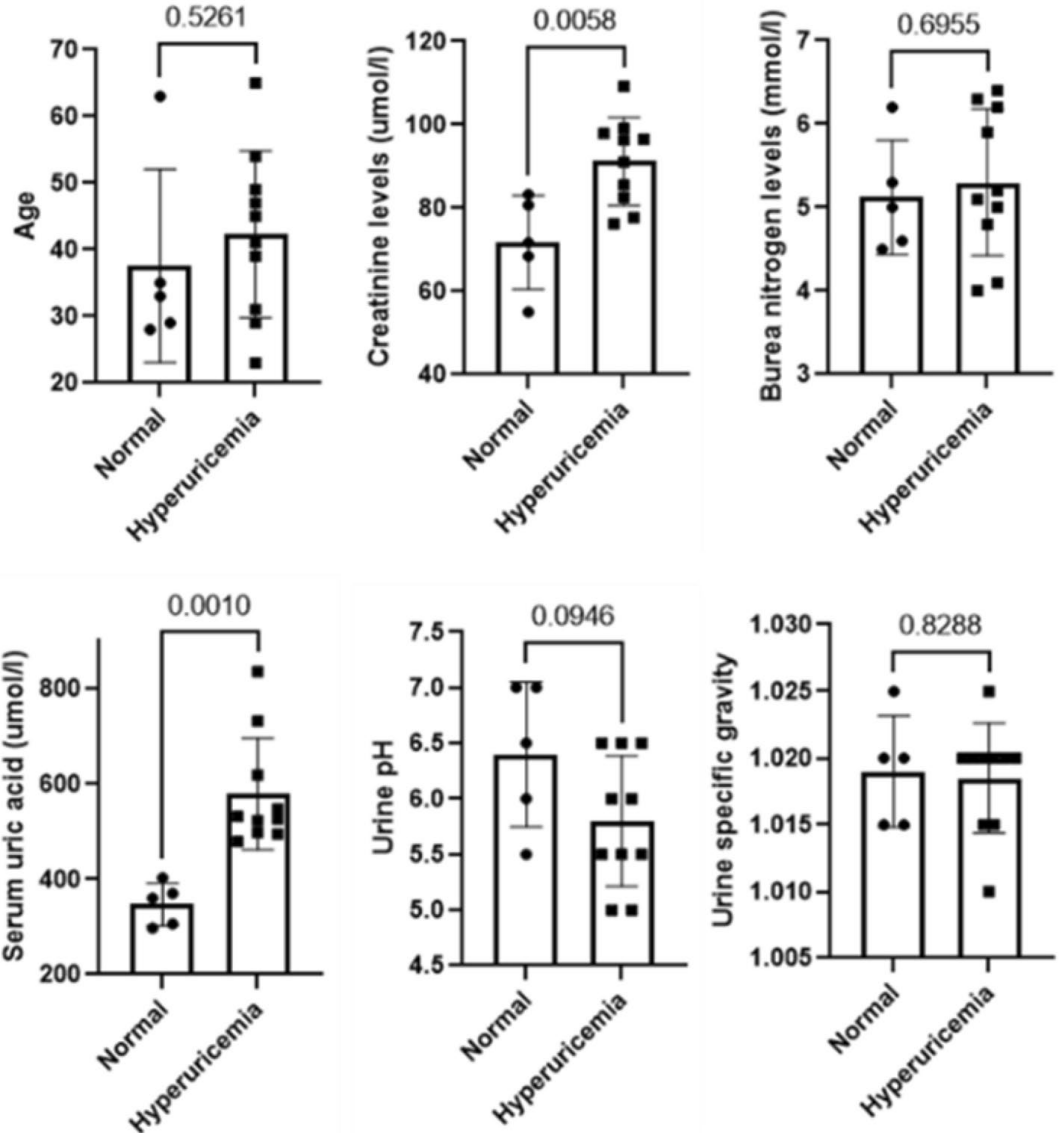
Comparison of the clinical characteristics of the enrolled subjects. Data are presented as means ± SD. Unpaired t-test p-values are shown above the bar chart respectively.

The hyperuricemia model was induced in mice by uricase inhibitor potassium oxonate (PO) as described previously^30^. Included were 3 controls and 3 hyperuricemia model mice. The uric acid levels in the serum of mice were detected to validate the success of the establishment of the hyperuricemia model (S-Fig. 1). The kidney samples were collected after the model was established and was sent for RNA sequencing using GPL23479 BGISEQ-500 (Mus musculus) Platforms. The RNA expression data were processed and normalized into log2 (TPM) (transcript per million) for analysis difference. T-test was used to analyse the difference between the two groups. A P-value of less than 0.05 was considered significant. This data set has been previously published as the GEO dataset series GSE186871 (https://www.ncbi.nlm.nih.gov/geo/query/acc.cgi?acc=GSE186871)^31^.

## Construction of hyperuricemia regulation network.

The differential expressed genes (DEG) between the hyperuricemia mouse and normal mouse kidney tissues were identified using a t-test. These genes were defined as hyperuricemia kidney DEG. An intersection analysis was conducted between the hyperuricemia KEGG targets and hyperuricemia kidney DEG. The resulting common genes were used to construct the male hyperuricemia regulation network using STRING^32^.

## Mendelian randomization analysis

Summary genetic association data on gout, herbal tea intake, coffee intake, and tea intake were obtained from a genome-wide association study from the FinnGen database (ID: finn-b-GOUT) and UKbiobank (ID: ukb-b-13344 and ukb-b-6066). The SNPs p-value threshold was set at 5e^−8^. We use clumping to prune SNPs with linkage disequilibrium Rsq > 0.001. The clumping distance was set at 10,000 kb. If a particular exposure SNP is not present in an outcome dataset, proxy SNPs with linkage disequilibrium Rsq > 0.8 were used instead. The minor allele frequency threshold for aligning palindromes was set at 0.3. For the allele harmonization issue, we attempted to align strands for palindromic SNPs. MR Egger, Weighted median, Inverse variance weighted, and Weighted mode method were used to estimate the effect respectively. Leave-one-out sensitivity analysis and funnel plot were used to assess the heterogeneity of the estimation. All the analyses were conducted using the TwoSampleMR version 0.5.5^33,34^.

## Results

### Clinical characteristics of the enrolled subjects

To avoid the impact of the female endocrine cycle, we only included male subjects in this study. The detailed clinical characteristics of the enrolled subjects are provided in S-Table 1. Based on the subjects indicators that we recorded at the time that we collected the samples, we compared the difference between normal donors and hyperuricemia patients. Results showed that normal donors and hyperuricemia patients have no significant differences in age, blood urea nitrogen levels, urine pH, and urine specific gravity. Although there is a trend that hyperuricemia patients have a lower urine pH, the difference was not statistically significant. On the other hand, the creatinine and serum uric acid levels were significantly higher in hyperuricemia patients compared to normal donors (Fig. 1).

### Identification of differential metabolites of hyperuricemia

**Figure 2:**
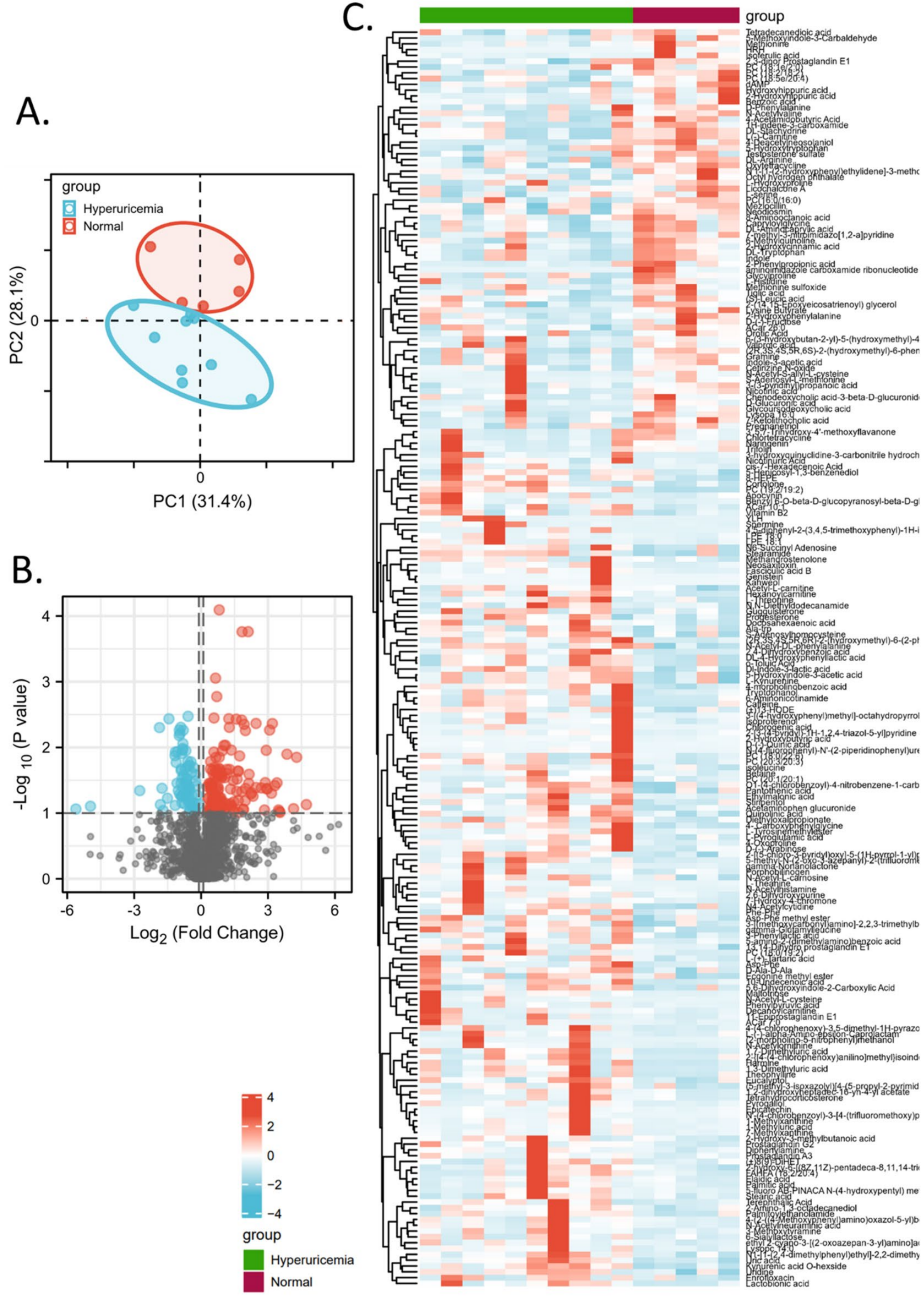
Identification of differential metabolites of hyperuricemia. Combine urine and serum data. (**A**) PCA plot showing the segregation of the participants based on the differential metabolite data. (**B**) Volcano plot of differential metabolites in hyperuricemia. Large size points represent significant metabolites. Red points represent up-regulated metabolites in patients compared to normal. Blue points represent down-regulated metabolites in patients compared to normal. (**C**) Heatmap of differential metabolites in hyperuricemia.

An untargeted metabolomic analysis using high-resolution MS was conducted to analyze serum and urine samples respectively. There were 480 peaks in positive and 257 peaks in negative ionization modes from serum samples that were detected. There were 341 peaks in positive and 659 peaks in negative ionization modes from urine samples that were detected. Representative traces of LC–MS/MS results are shown in S-Fig. 2. After the combination of all these identified metabolites with a database matching, 1737 metabolites were identified. Data quality in the metabolic analysis and the intrinsic metabolic variations were evaluated by a PCA analysis (Fig. 2A). Among these metabolites, 227 differential metabolites were identified as significantly differential metabolites in serum and urine between normal donors and hyperuricemia patients (Fig. 2B), including 118 metabolites hitting chemicals in HMDB, PubChem, and KEGG databases (“supplementary table(metabolite mapping)”). The quantitative results of these metabolites were displayed in a heatmap (Fig. 2C).

### Enrichment analysis of the differential metabolites

**Figure 3:**
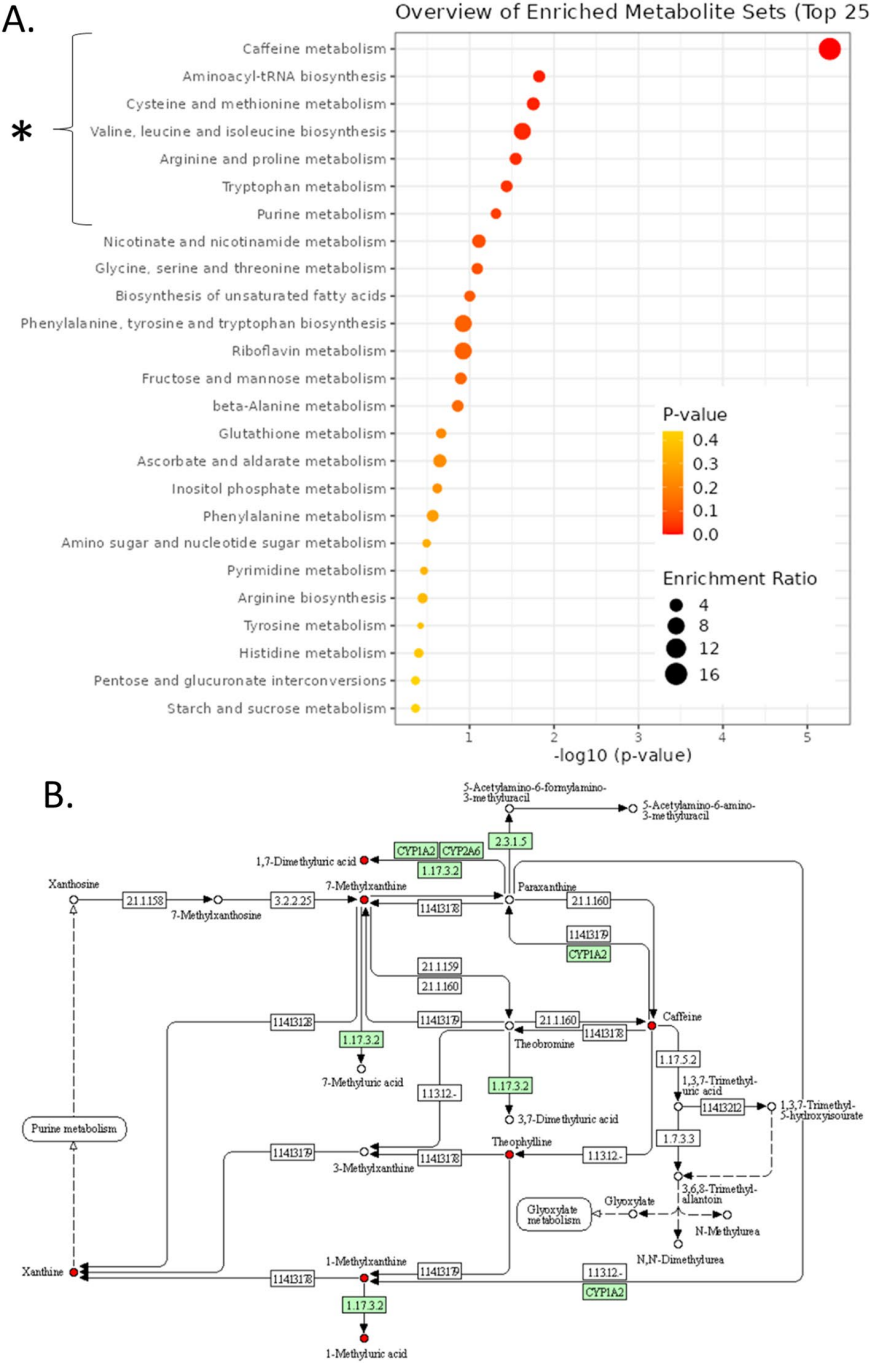
KEGG metabolic pathways enrichment. (**A**) Bubble chart of the KEGG metabolic pathways enrichment (*p < 0.05). (**B**) Mapping of hyperuricemia differential metabolites in KEGG “Caffeine metabolism” reference pathways. Hit metabolites were highlighted in red. The genes in green were human genes. KEGG pathway mapper (00232 11/30/18) were used to create the map. (https://www.genome.jp/kegg/).

**Figure 4:**
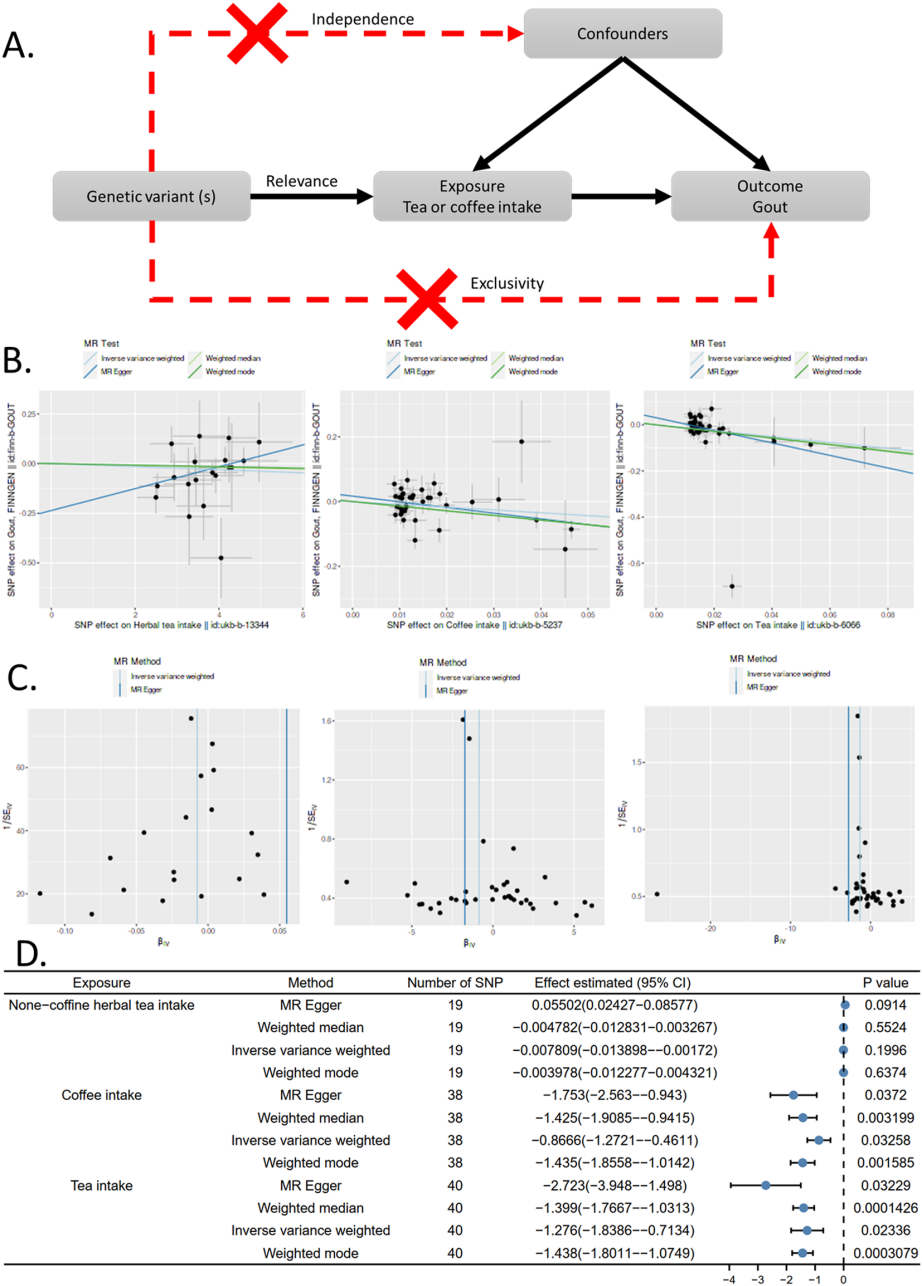
Mendelian randomization analysis of the association between caffeine-containing drinks and gout risk. Coffee and tea were analyzed respectively. None-caffeine herbal tea was used as a negative control. (**A**) Directed acyclic diagram demonstrating the core assumptions of Mendelian randomization in this study. (**B**) SNP effects on the outcome are plotted against SNP effects on the exposure. (**C**) Funnel plot to assess heterogeneity. (**D**) Mendelian randomization analysis results. MRBASE (1.4.3 8a77eb) was used to conducted the analysis and plotting.

**Figure 5:**
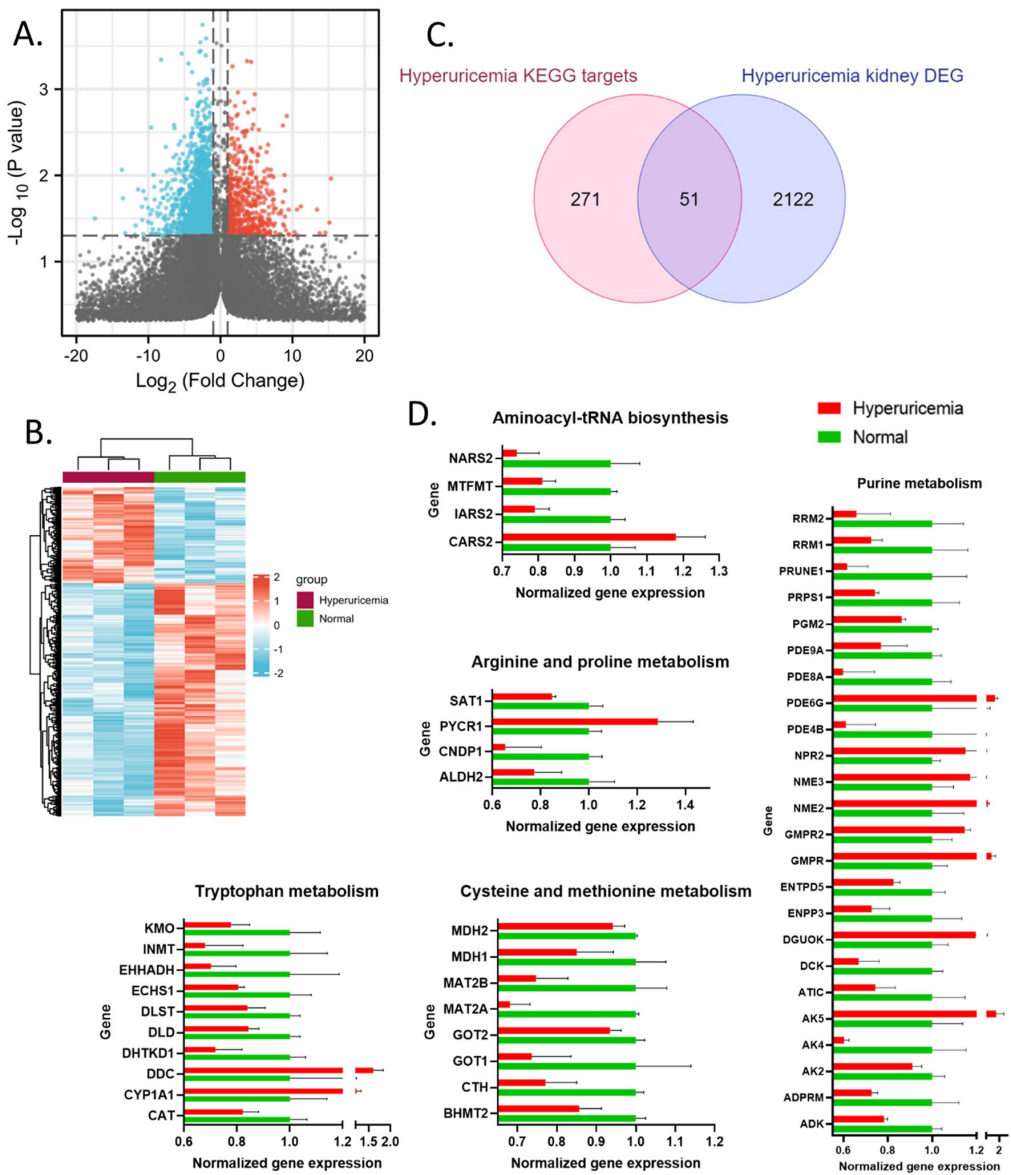
Identification of critical kidney genes involved in hyperuricemia. (**A**) Volcano plot of the differential expressed genes (DEG) between the hyperuricemia mouse and normal mouse kidney tissues. These genes were defined as hyperuricemia kidney DEG. Plot with R (4.2.1) and ggplot2(3.3.6). (**B**) Heatmap of the hyperuricemia kidney DEG. (**C**) Intersection analysis between the hyperuricemia KEGG targets (genes in pathways where the hit metabolites are significantly enriched) and hyperuricemia kidney DEG. (**D**) Normalized expression (log_2_(TPM + 0.001)) of critical kidney genes involved in hyperuricemia (means ± SD, all *p* < 0.05).

**Figure 6:**
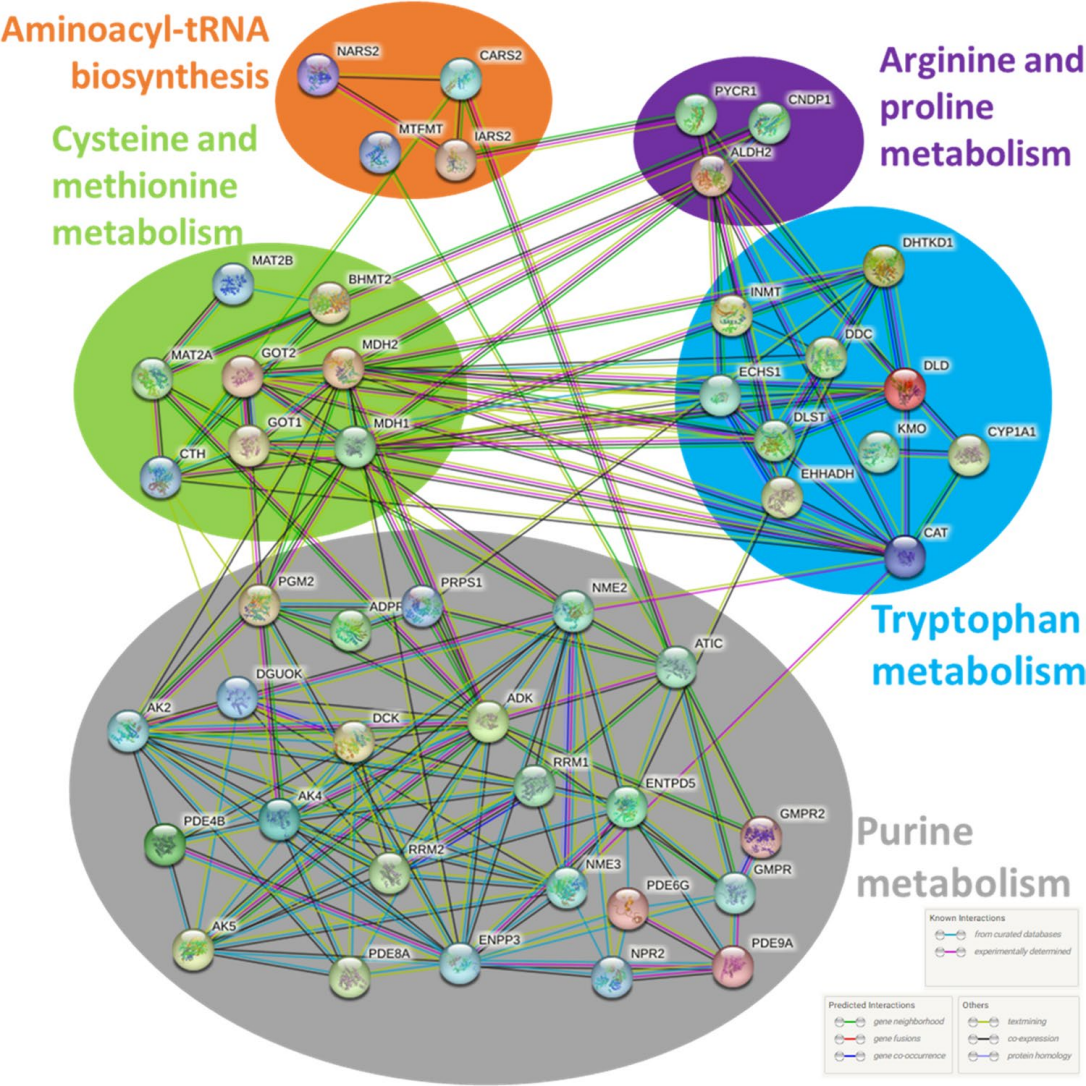
Hyperuricemia regulation protein network. Generated with STRING tool (https://string-db.org/).

The significantly different metabolites were used for enrichment analyses. The first enrichment analysis we conducted was with the KEGG database. Results showed that these metabolites were significantly enriched in “Caffeine metabolism”, “Aminoacyl-tRNA biosynthesis”, “Cysteine and methionine metabolism”, “Valine, leucine, and isoleucine biosynthesis”, “Arginine and proline metabolism”, “Tryptophan metabolism”, and “Purine metabolism” (Fig. 3A). We collected 347 genes involved in these pathways and defined them as hyperuricemia KEGG targets for the subsequent analysis. Surprisingly, the top enriched KEGG pathway was “Caffeine metabolism”, which was strikingly significant. In fact, when we analyzed the serum and urine results separately, “Caffeine metabolism” was the top enriched in both analyses (S-Fig. 3). Hence, we mapped the metabolites to the KEGG “Caffeine metabolism” pathway for a better display of the potential role of the “Caffeine metabolism” pathway in hyperuricemia. As shown in the pathway Fig. 3B, 7 nodes were hit including 5 hub nodes. In addition, we also conducted an enrichment analysis using the SMPDB. Results showed that these metabolites were significantly enriched in “Methylhistidine Metabolism”, “Caffeine Metabolism”, “Methionine Metabolism”, “Betaine Metabolism”, and “Tryptophan Metabolism” and we also mapped the metabolites to the top two pathways in the SMPDB (S-Figs. 4–6). Details were provided in the file “supplementary table (SMPDB)”.

### Mendelian randomization analysis of the association between caffeine-containing drinks and gout risk

Our metabolites analysis suggested that caffeine metabolism might affect hyperuricemia, hence, to explore the potential association between caffeine metabolism and hyperuricemia, we conducted a Mendelian randomization analysis of the association between caffeine-containing drinks and gout risk. Gout is caused by a condition known as hyperuricemia, where there is too much uric acid in the body. Therefore, gout is a symptom of severe hyperuricemia. Coffee and tea are the two most popular caffeine-containing drinks. Gout is the major symptom of hyperuricemia, but because of the interference of confounders, the association between tea or coffee intake and gout remains unclear. In this study, we used genetic variants as the instruments to estimate the effect of tea or coffee intake on gout. We also included herbal tea intake as a comparison group. In the Mendelian randomization, we assumed that a) the genetic variant strongly associates with the tea or coffee intake, b) there is no confounding of the genetic variant-outcome gout, and c) the genetic variant only affects gout through the tea or coffee intake (Fig. 4A). In the Mendelian randomization analysis, we conducted four algorithms to estimate the association between caffeine-containing drinks and gout risk and used funnel plots (Fig. 4C) to assess heterogeneity. SNP effects on the outcome are plotted against SNP effects on the exposure (Fig. 4B). Results showed that both coffee intake and tea intake were significantly associated with gout with all four algorithms, but the herbal tea intake did not have significant effects on gout (Fig. 4D). These data further supported the association between caffeine metabolism and hyperuricemia.

### Construction of hyperuricemia regulation network

It is well known that hyperuricemia is closely associated with chronic kidney disease. This disease leads to hyperuricemia due to decreased urinary excretion of UA. Hyperuricemia also may induce kidney dysfunction and contribute to chronic kidney disease progression through a number of potential mechanisms^35^. To identify the critical kidney genes involved in hyperuricemia, we jointly analyzed differential human metabolites of hyperuricemia and differential expressed genes (DEG) in the kidney in the hyperuricemia mouse model. The hyperuricemia model was induced in mice by the uricase inhibitor potassium oxonate and the DEGs in the kidney between normal control and hyperuricemia animals were identified from the RNA-sequencing results (Fig. 5A). We identified 2173 genes, which were defined as hyperuricemia kidney DEG, and their expressions were displayed in a heatmap (Fig. 5B and “supplementary table(DEG in mice)”). An intersection analysis between the hyperuricemia KEGG targets (previously defined in this study as the genes in pathways where the hit metabolites are significantly enriched) and hyperuricemia kidney DEGs was conducted to identify critical kidney genes involved in hyperuricemia. Results identified 51 genes involved in 5 KEGG pathways (Fig. 5C), including 4 genes in “Aminoacyl-tRNA biosynthesis”, 4 genes in “Arginine and proline metabolism”, 10 genes in “Tryptophan metabolism”, 5 genes in “Cysteine and methionine metabolism”, and 24 genes in “Purine metabolism” pathway. The expression of these genes in animal kidneys were shown in Fig. 5D. Based on these genes, we constructed a hyperuricemia regulation network using the protein–protein-interaction network function of the STRING (Fig. 6). This network illustrates the complex interrelationships among the genes implicated in hyperuricemia kidney disease. It reveals that, despite the fact that these genes are in different pathways, there is still a considerable amount of interaction that occurs across them. This provides a comprehensive view of the regulation network of hyperuricemia kidney disease, which can be used as a reference for future research.

## Discussions

Hyperuricemia is a relatively less studied disease. It is known by most people as the cause of gout. For the past decade, high-throughput metabolomics analysis was regarded as an emerging field. Metabolomics analysis has developed with a higher sensitivity, a lower cost, and a more comprehensive metabolite database. The application of metabolomics analysis has been induced in the hyperuricemia study in recent years. So far, only a few studies have reported metabolomics analysis using samples associated with hyperuricemia. Studies have reported serum metabolomics analysis^23–25^ as well as animal model urine metabolomics analysis^26^. Yet, no study has been done to combine metabolomics analysis of serum and urine from the same patients at the same time. Compared to the previous studies which analyzed blood or urine solely, the joint analysis of blood and urine samples in this study provided a more comprehensive observation of the alteration in metabolism.

A previous serum metabolomics analysis identified hyperuricemia-associated pathways and hyperuricemia-characteristic biomarkers of hyperuricemia and gout, which included over 300 participants and drew a rather reliable conclusion^24^. Another study also reported a serum metabolomics analysis of hyperuricemia, which included 20 patients and 20 healthy donors^23^. Compared to these previous studies, one of the limitations of the present study is that the number of samples included was relatively low, hence, this study can only be regarded as a pilot study rather than a conclusive research study. In this study, we want to identify the most distinguished differential metabolisms. We did not conduct power analysis because power analysis is only applicable to targeted metabolic analysis. However, we did an un-targeted analysis in which we are not sure which metabolisms can be identified, thus, it is impossible to conduct power analysis before the experiments. We admit that, with such a small case number, we should have missed a lot of differential metabolisms that are only slightly different between the patient group and the normal group. In addition, to enable relatively stable results and consistency within a group, unlike the previous studies, this study only included male participants. Hyperuricemia has been found to be significantly different in male and female populations^36^, thus, gender is an interfering factor that might be detrimental to this kind of study. Moreover, for females, the blood samples and urine samples might be impacted by their menstrual cycle. The PCA analysis of the metabolites showed an obvious separation between hyperuricemia and the control group, which indicated consistent metabolite results within each group. Despite the low number of samples, which would result in there being fewer significant metabolites identified, by combining metabolites identified in serum and urine, we believe that although some pathways with trivial effects might be missed, the major pathways involved in hyperuricemia can be identified in the enrichment analysis.

A previous study using urinary metabolomics to evaluate the effect of hyperuricemia on the kidney discovered that inflammation induced by the interleukin 6/signal transducer and activator of the transcription 3 signalling pathway participated in hyperuricemia-induced kidney injury^26^. However, this is an animal study with urine samples only, which were different from our study. In addition, a previous study using untargeted metabolomics to analyze serum from patients suggested that hyperuricemia was associated with metabolic pathways involved in glycerophospholipid metabolism, sphingolipid metabolism, arachidonic acid metabolism, linoleic acid metabolism, phenylalanine metabolism, phenylalanine, tyrosine and tryptophan biosynthesis, and a-linolenic. This study was in line with our results that tryptophan metabolism might be involved in hyperuricemia^23^. Another previous study which also analyzed serum from hyperuricemia patients revealed that hyperuricemia is primarily associated with 4 pathways: (1) arginine and proline metabolism; (2) ascorbate and aldarate metabolism; (3) taurine and hypotaurine metabolism; and (4) alanine, aspartate, and glutamate metabolism. In our study, we also found that arginine and proline metabolism is one of the involved in hyperuricemia patients^23^. However, their study did not analyse urine samples which might result from the differences in our findings. Additionally, this study identified a novel form of caffeine metabolism, which had not been observed in previous studies.

Age is a critical factor for hyperuricemia. A meta-analysis based on the prevalence of hyperuricemia in China concluded that the prevalence of hyperuricemia significantly increases after 30 years for men and 50 years for women^37^. However, in this study, the youngest hyperuricemia patient was only 23. This reminded us that hyperuricemia should be prevented as young as possible. It has been discussed whether tea or coffee can prevent hyperuricemia. A surprising finding of this study is that the hyperuricemia metabolites were significantly enriched in “Caffeine metabolism” with a very striking significance and 5 metabolites were located at the hub nodes in the KEGG “Caffeine metabolism” pathway. Although we failed to collect information about the coffee, tea, and caffeine intake of the subjects, all the subjects were from south China where most people drink tea. It is unsure whether the significant caffeine metabolism enrichment was associated with the caffeine in their tea intake. Nevertheless, our study brought in a potential impact of caffeine on hyperuricemia. To date, a number of clinical studies have reported the association between caffeine and hyperuricemia. Previous analysis suggested that coffee, tea, and caffeine consumption were not associated with the risk of hyperuricemia in a Korean population cohort^38^. A study in the US reported that coffee, but not tea, intake leads to a lower serum uric acid level and hyperuricemia frequency^39^. A randomized within-subject experimental study reported that decaffeinated coffee caused a significant decrease in serum uric acid, and caffeinated coffee might cause an increase in serum uric acid in healthy people^40^. Another Korean study, a systematic review and meta-analysis, suggested that moderate coffee consumption might prevent hyperuricemia and gout^41^. Overall, our findings further supported the association between caffeine intake and hyperuricemia. In addition, our Mendelian randomization analysis of the association between caffeine-containing drinks and gout risk further supports this relationship. Yet, limited to the data availability, the Mendelian randomization analysis was conducted with genetic data collected from Europeans, which is different from the patients from whom we collected metabolomics data. More studies are required in the future to further explore the potential mechanism underlying this association.

The kidney has been thought to be the major organ associated with hyperuricemia. In fact, the pathological changes in the kidney might lead to hyperuricemia, but the hyperuricemia itself might impact the kidney in turn. For example, a study reported that hyperuricemia resulted in kidney damage via inducing autophagy and NLRP3-mediated inflammation^42^. In the present study, we identified the differentially expressed genes in the kidney, however, it is unsure if the alteration of the gene expression was a cause or a result of hyperuricemia. In addition, the number of mice was relatively low, thus the results might be subject to false positives. Overall, even though we identified the altered gene correctly, the mouse model was still different from the human model. The metabolites enrichment analysis was based on human databases, which might be slightly different from mouse databases, therefore, the joint analysis of the human metabolomics and a mouse model forms a pilot study. Nevertheless, given that human kidney samples from hyperuricemia are difficult to collect and mice are similar to humans in most genes (that is why we use the mouse model to mimic human diseases), the hyperuricemia regulation network we constructed is still valuable as a reference for future studies.

## Conclusion

This study suggested a potential association between caffeine metabolism and hyperuricemia and constructed a hyperuricemia regulation protein network in the kidney for future reference.

## Supplementary Information

Supplementary Information 1.

Supplementary Information 2.

Supplementary Information 3.

Supplementary Information 4.

## Data Availability

Raw data are provided by the corresponding author with a reasonable request. GEO dataset series can be accessed from GSE186871 (https://www.ncbi.nlm.nih.gov/geo/query/acc.cgi?acc=GSE186871).
