# Supplementary material for: Exploring the mechanism underlying hyperuricemia using comprehensive research on multi-omics
Source: Sci Rep. 2023 May 3;13:7161. doi: 10.1038/s41598-023-34426-y (PMC10156710; doi:10.1038/s41598-023-34426-y)
Supplement: Supplementary file 4 — Supplementary Information 4. [file 41598_2023_34426_MOESM4_ESM.docx]

**Supplimentary materials**


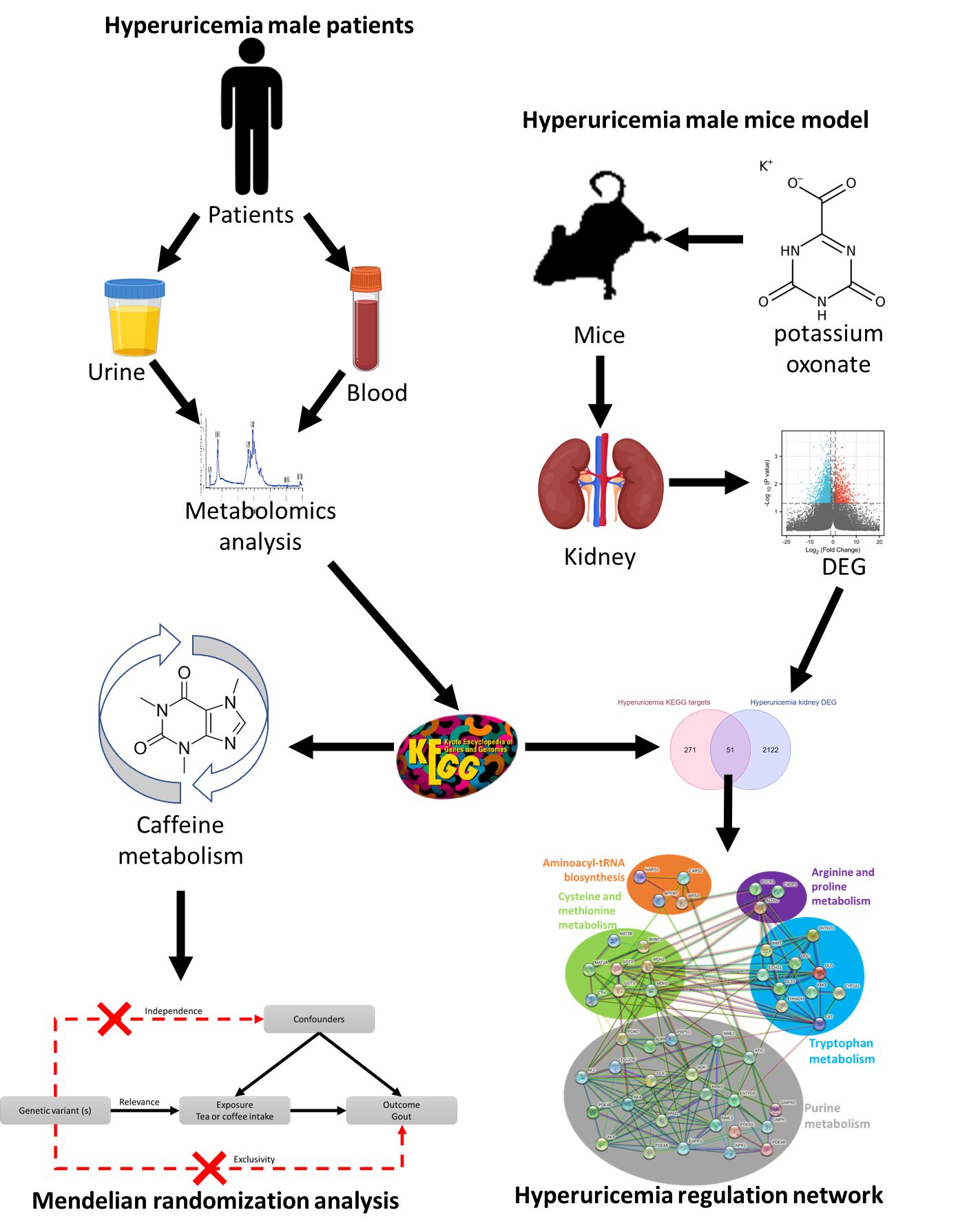


**S-Figure.** Graphical abstract

**S-Table 1.** Clinical characteristics of the enrolled subjects.

| Group | Age | Creatinine levels (µmol/l) | Blood urea nitrogen levels (mmol/l) | Serum uric acid (μmol/l) | Urine pH | Urine specific gravity |
| --- | --- | --- | --- | --- | --- | --- |
| Normal | 35 | 71.7 | 4.5 | 370.2 | 7 | 1.015 |
| Normal | 33 | 68.4 | 4.6 | 296.9 | 6 | 1.02 |
| Normal | 63 | 55 | 5.3 | 306.7 | 7 | 1.015 |
| Normal | 29 | 83.2 | 5,9 | 359.9 | 6.5 | 1.02 |
| Normal | 28 | 80.7 | 6.2 | 402.5 | 5.5 | 1.025 |
| Hyperuricemia | 31 | 109.2 | 6.3 | 497.9 | 6 | 1.02 |
| Hyperuricemia | 54 | 76.2 | 6.4 | 523 | 5.5 | 1.02 |
| Hyperuricemia | 49 | 97.9 | 5.1 | 479.1 | 6.5 | 1.02 |
| Hyperuricemia | 47 | 96.5 | 4.8 | 494.9 | 6 | 1.02 |
| Hyperuricemia | 45 | 82.6 | 4 | 531.8 | 6.5 | 1.01 |
| Hyperuricemia | 23 | 77.7 | 4.1 | 835.7 | 5.5 | 1.015 |
| Hyperuricemia | 41 | 96.3 | 5 | 618.6 | 5 | 1.025 |
| Hyperuricemia | 65 | 99.1 | 5.2 | 548 | 6.5 | 1.015 |
| Hyperuricemia | 39 | 91,6 | 6.2 | 526.4 | 5 | 1.02 |
| Hyperuricemia | 29 | 85.5 | 5.9 | 731.5 | 5.5 | 1.02 |
|  |  |  |  |  |  |  |

**Supplementary table(metabolite mapping).** This table listed the identified metabolites that match the databases.

**Supplementary table (SMPDB).** This table summarized the enrichment results of differential metabolites.

**Supplementary table(DEG in mice).** This table included the differential expression analysis results of Hyperuricemia mice.


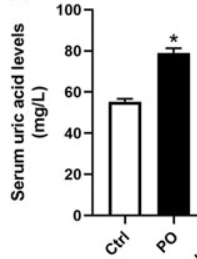


**S-Figure 1.** The uric acid level in hyperuricemia model. Ctrl: control mice, Po: potassium oxonate mice. The uric acid levels in the serum of mice were detected to validate the success of the establishment of the hyperuricemia model. This figure is extracted from the previous papers that published dataset series GSE186871.


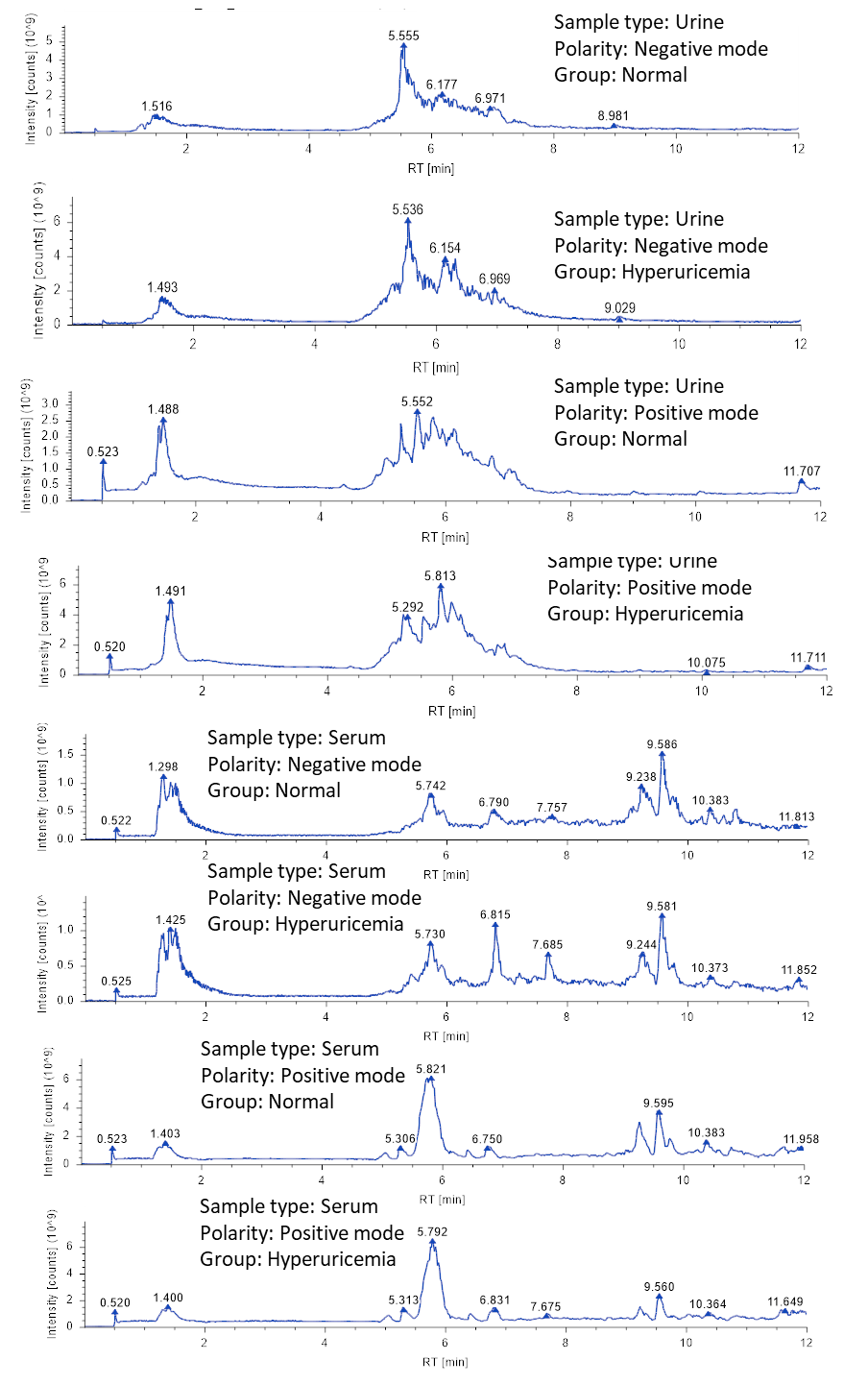


**S-Figure 2.** Representative traces of LC-MS/MS. The top identified peaks were labeled.


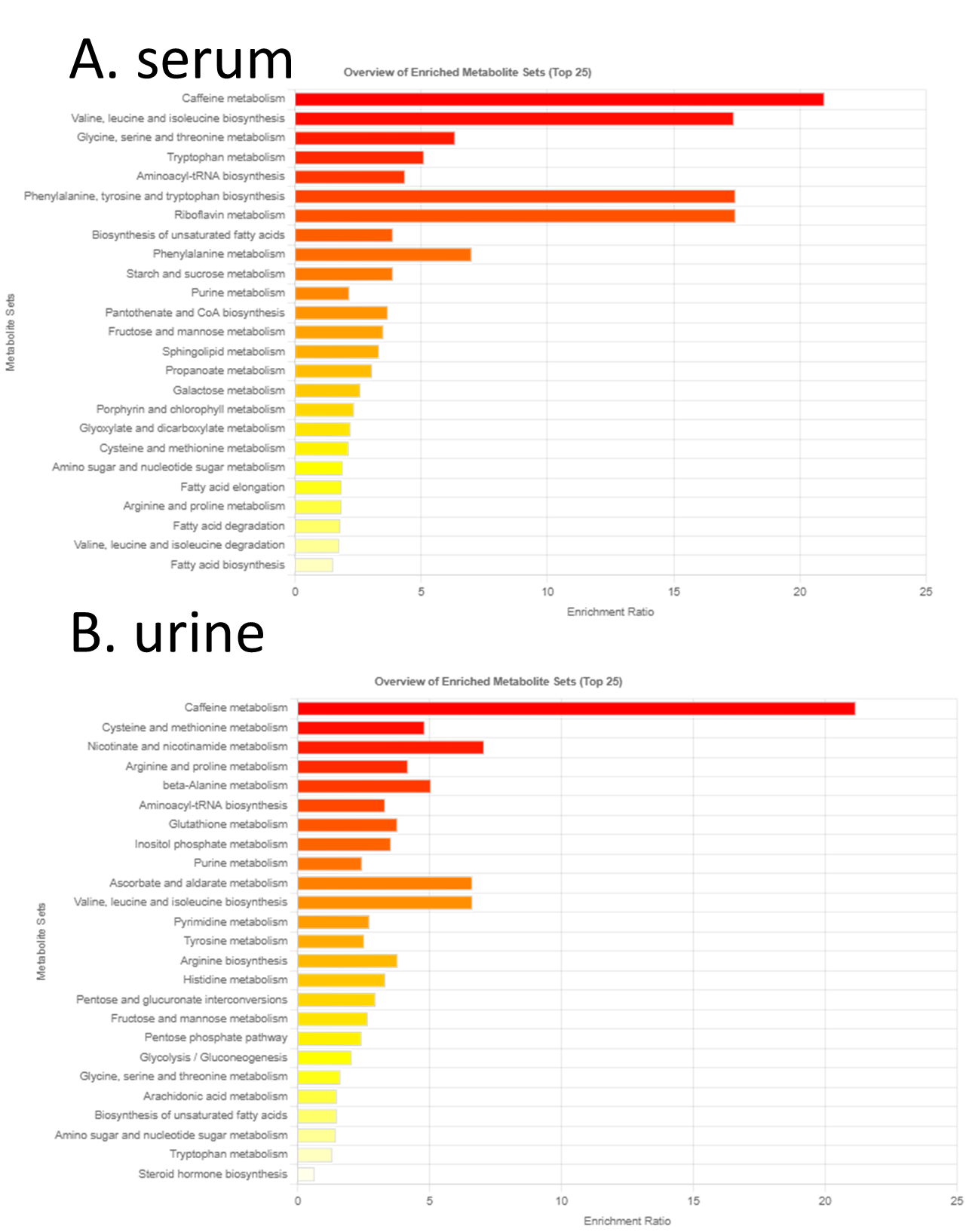


**S-Figure 3.** KEGG metabolic pathways enrichment for serum and urine samples respectively.


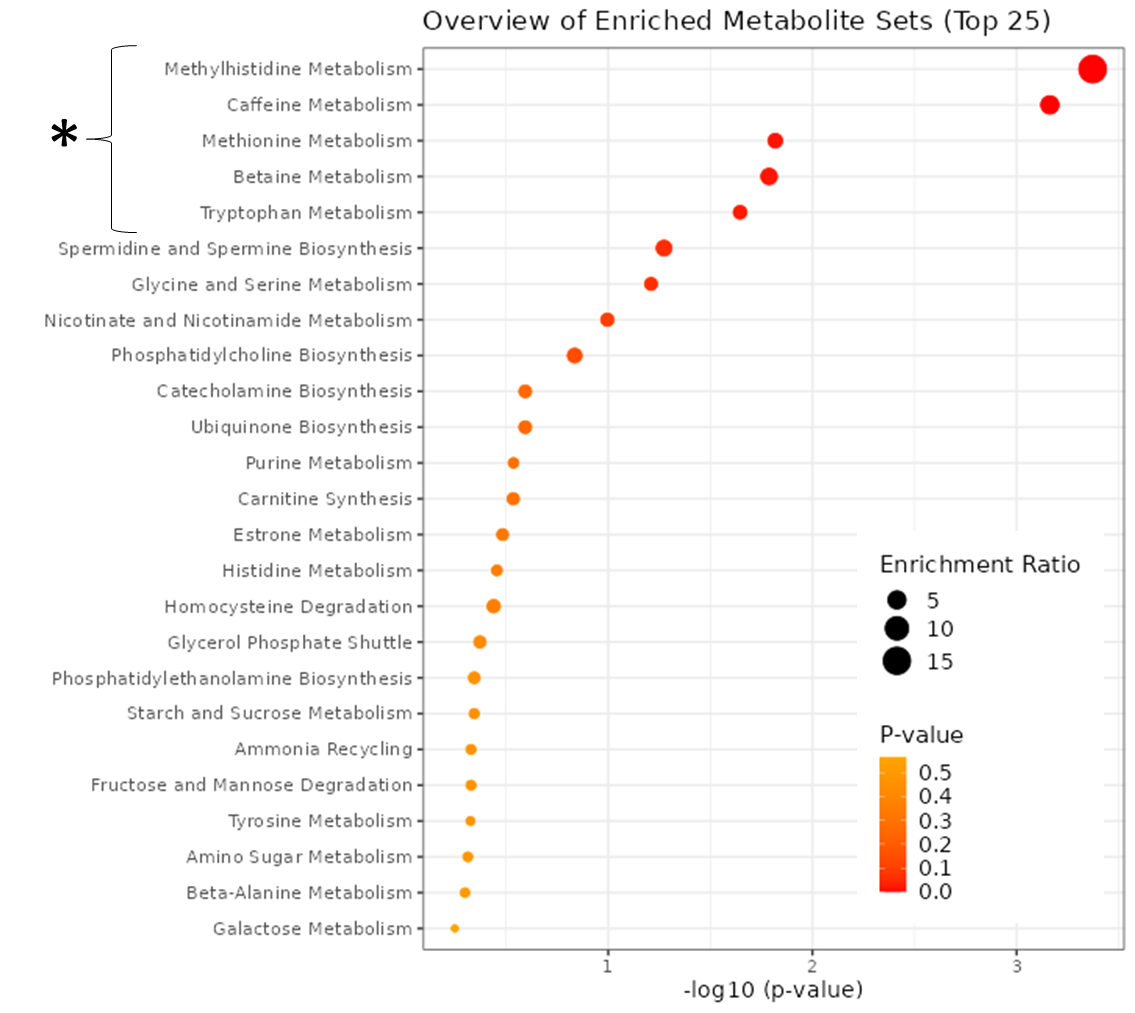


**S-Figure 4.** Enrichment analysis in 99 metabolite sets based on normal human metabolic pathway (*p<0.05).


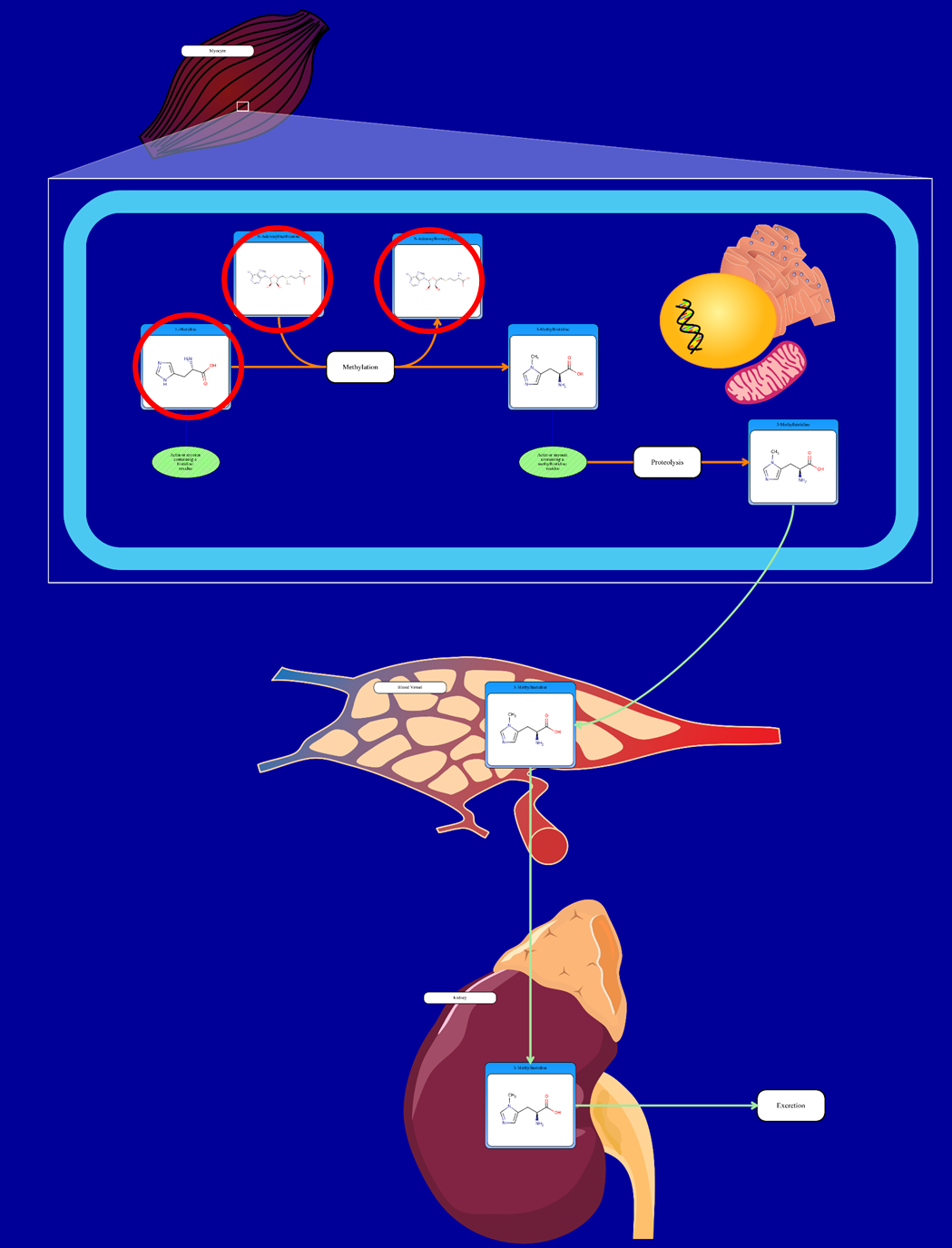


**S-Figure 5.** Pathway mapping of methylhistidine metabolism in SMPDB. Hit metabolites were highlighted in red circles. High resolution pathway plot can be accessed at https://www.smpdb.ca/view/SMP0000715.


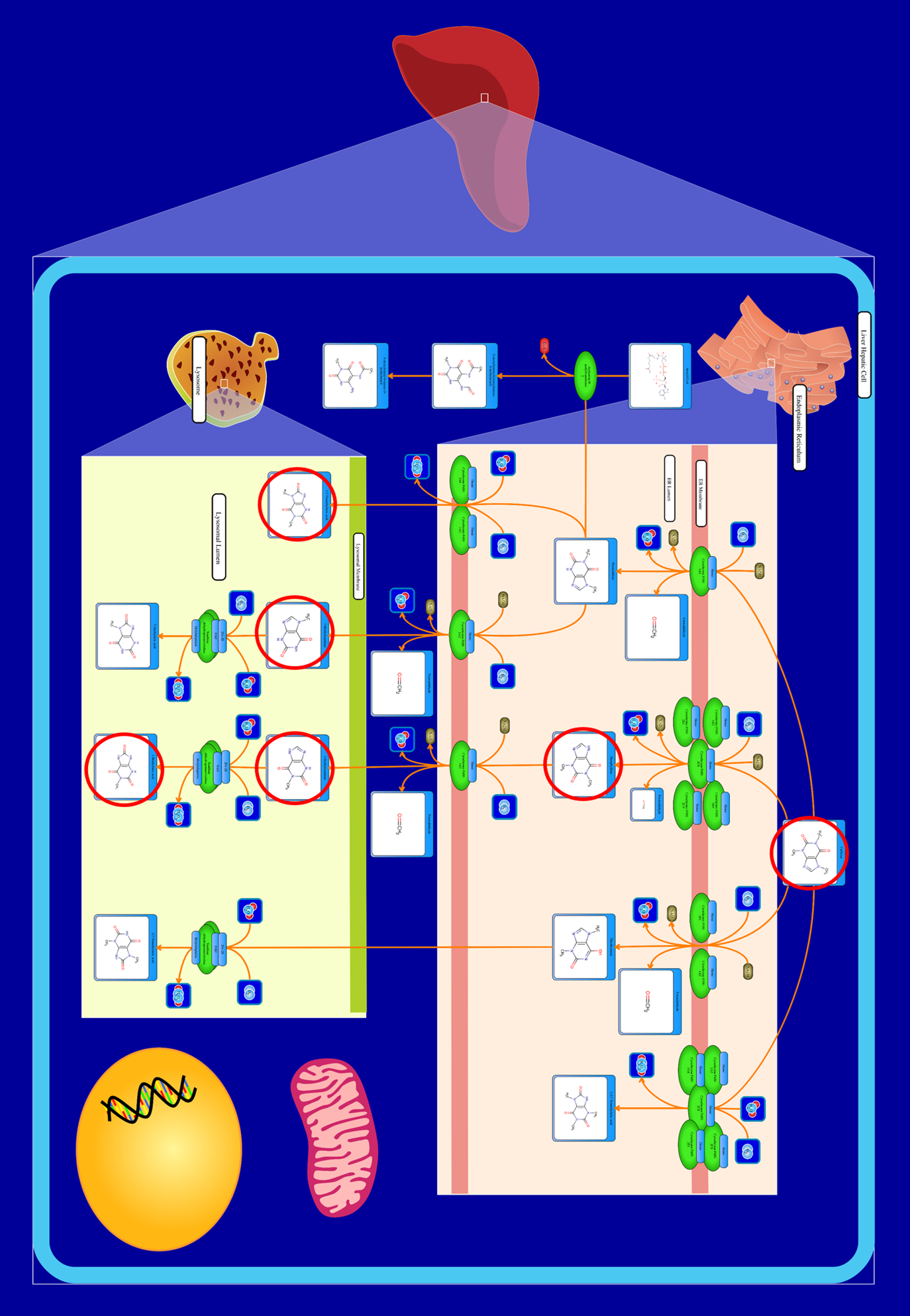


**S-Figure 6.** Pathway mapping of caffeine metabolism in SMPDB. Hit metabolites were highlighted in red circles. High resolution pathway plot can be accessed at https://www.smpdb.ca/view/SMP0000028.
